# Spatial variation in gene expression of Tasmanian devil facial tumors despite minimal host transcriptomic response to infection

**DOI:** 10.1186/s12864-021-07994-4

**Published:** 2021-09-27

**Authors:** Christopher P. Kozakiewicz, Alexandra K. Fraik, Austin H. Patton, Manuel Ruiz-Aravena, David G. Hamilton, Rodrigo Hamede, Hamish McCallum, Paul A. Hohenlohe, Mark J. Margres, Menna E. Jones, Andrew Storfer

**Affiliations:** 1 School of Biological Sciences, Washington State University, Pullman, Washington USA; 2 Department of Integrative Biology, University of California, Berkeley, California USA; 3 Department of Microbiology & Immunology, Montana State University, Bozeman, MT USA; 4 School of Natural Sciences, University of Tasmania, Hobart, Tasmania Australia; 5 CANECEV, Centre de Recherches Ecologiques et Evolutives sur le Cancer, 34090 Montpellier, France; 6 Environmental Futures Research Institute, Griffith University, Nathan, Queensland Australia; 7 Department of Biological Sciences, Institute for Bioinformatics and Evolutionary Studies, University of Idaho, Moscow, ID USA; 8 Department of Integrative Biology, University of South Florida, Tampa, Florida USA

**Keywords:** Transmissible cancer, Host-pathogen coevolution, Wildlife disease, Population transcriptomics

## Abstract

**Background:**

Transmissible cancers lie at the intersection of oncology and infectious disease, two traditionally divergent fields for which gene expression studies are particularly useful for identifying the molecular basis of phenotypic variation. In oncology, transcriptomics studies, which characterize the expression of thousands of genes, have identified processes leading to heterogeneity in cancer phenotypes and individual prognoses. More generally, transcriptomics studies of infectious diseases characterize interactions between host, pathogen, and environment to better predict population-level outcomes. Tasmanian devils have been impacted dramatically by a transmissible cancer (devil facial tumor disease; DFTD) that has led to widespread population declines. Despite initial predictions of extinction, populations have persisted at low levels, due in part to heterogeneity in host responses, particularly between sexes. However, the processes underlying this variation remain unknown.

**Results:**

We sequenced transcriptomes from healthy and DFTD-infected devils, as well as DFTD tumors, to characterize host responses to DFTD infection, identify differing host-tumor molecular interactions between sexes, and investigate the extent to which tumor gene expression varies among host populations. We found minimal variation in gene expression of devil lip tissues, either with respect to DFTD infection status or sex. However, 4088 genes were differentially expressed in tumors among our sampling localities. Pathways that were up- or downregulated in DFTD tumors relative to normal tissues exhibited the same patterns of expression with greater intensity in tumors from localities that experienced DFTD for longer. No mRNA sequence variants were associated with expression variation.

**Conclusions:**

Expression variation among localities may reflect morphological differences in tumors that alter ratios of normal-to-tumor cells within biopsies. Phenotypic variation in tumors may arise from environmental variation or differences in host immune response that were undetectable in lip biopsies, potentially reflecting variation in host-tumor coevolutionary relationships among sites that differ in the time since DFTD arrival.

**Supplementary Information:**

The online version contains supplementary material available at 10.1186/s12864-021-07994-4.

## Background

Identification of the processes underlying cancer development as well as those associated with heterogeneity in its progression is critical for predicting both individual and population-level outcomes [1, 2]. Measuring relative levels of gene expression has been key for identifying genes and biological pathways associated with cancers and heterogeneity in cancer phenotypes [3–5]. For example, gene expression studies have shown regulatory dysfunctions associated with tumorigenesis [6], identified expression profiles associated with therapy resistance and poor prognoses [7–9], and provided insights into interactions between tumor cells and the immune system [10]. Similarly, gene expression analyses are frequently used to understand how organisms respond to environmental stressors, such as thermal stress [11], pollutants [12], and infections [13]. Variation in gene expression among individuals can also indicate how biotic and abiotic pressures underlie population-level responses. For example, temperature-dependent susceptibility of salamanders to *Batrachochytrium dendrobatidis* (Bd) is driven by temperature-mediated shifts in innate versus adaptive immune gene expression, enabling predictions of Bd effects on amphibian communities under different climate change scenarios [14]. The fields of oncology and wildlife disease intersect in the case of transmissible cancers, which are clonal, transmissible tumors that are spread among individuals by the transfer of cancerous cells [15].

Transmissible cancers are rare; the two most well-known examples are canine transmissible venereal tumor (CTVT) and Tasmanian devil facial tumor disease (DFTD), with other examples found in Syrian hamsters and mollusks [15, 16]. Whereas CTVT has relatively benign effects on its hosts both at the individual and population levels, DFTD is an aggressive, highly virulent pathogen with nearly 100% case fatality rate and has had devastating effects on Tasmanian devil populations [17–19]. Since its discovery in 1996, DFTD has spread throughout the entire geographic range of the Tasmanian devil, leading to rapid population declines exceeding 80% [20, 21]. Likely derived from a Schwann (peripheral nerve) cell cancer in a female devil [22], mutations leading to downregulation of MHC class I expression in the tumor [23, 24], coupled with potential natural killer cell dysfunction in devils [25], enabled DFTD to evade host allograft rejection and become transmissible [26, 27]. DFTD is transmitted via biting, a fundamental behavior in devil social interactions [28, 29], with DFTD tumors manifesting externally and predominantly around the mouth and face. Following visible presentation of tumor growth, progression is rapid, leading to death within 12 months [30].

Despite observations of frequency-dependent transmission leading to early predictions of devil extinction [18, 31], recent epidemiological models incorporating individual variation in pathogen load suggest that extinction is an unlikely outcome [32, 33]. Inter-individual variation in tumor growth rates and latency periods, together with a lack of vertical transmission, often enables females to survive through their first breeding season, allowing populations to persist at low densities despite high DFTD prevalence [32]. Female responses contributing to this success include increased precocial breeding and fecundity in the first breeding season in DFTD-affected populations, as well as tolerance of DFTD that manifests in a slower loss of body condition in DFTD-infected females relative to males [34–36].

Recent genomic evidence suggests rapid evolutionary responses of devils to DFTD. Genomic regions putatively associated with immune response, cancer resistance, and behavior appear to be evolving within as few as four generations (approx. 8 years) under positive selection in DFTD-affected populations [37, 38]. Females may also be adapting to DFTD through greater tolerance, with several associated genes of large effect detected in a genome-wide association study [39]. Although putative functions of candidate genes identified in these studies suggest biological functions that may underlie variation in host fitness, mechanistic differences in response to infection between sexes remain unclear. Additionally, isolated cases of spontaneous tumor regression have been observed in the field, the molecular underpinnings of which appear to be in regulatory regions because no non-synonymous substitutions have been found in either the devils or tumors [40, 41]. Further, phenotypic responses in devil populations following DFTD arrival have been observed within one or two generations [36], suggesting existing plasticity and not a purely adaptive response.

Studies comparing transcriptomic and genomic variation are ideally suited for elucidating the molecular basis for variation in devil responses to DFTD. However, previous gene expression studies of DFTD have aimed to identify cell type of origin [22] or targeted specific sets of immune-related genes, primarily in laboratory-cultured DFTD cell lines [e.g., 41–44]. Thus, there is a need to understand variation in both host and DFTD tumor gene expression in natural populations, particularly with respect to observed variation in DFTD-associated impacts on hosts.

To investigate the role of gene expression variation in manifestation of DFTD, we performed a population transcriptomics study of wild Tasmanian devils from DFTD-affected populations. We sequenced mRNA from normal tissues in both healthy and DFTD-infected devils, as well as tissues from DFTD tumors, and tested predictions of differential gene expression with respect to sex and population that could explain individual- and population-level responses to disease. We predicted that 1) similar to other types of cancer, DFTD exhibits gene expression that is distinct from normal host tissues, 2) the severe disease associated with DFTD infection induces significant responses in infected hosts as evidenced by expression differentiation between infected and uninfected hosts, 3) previously observed differences in DFTD tolerance between host sexes will be reflected in inter-sex variation in gene expression, and 4) spatial genetic variation in both devils and DFTD produces variation in gene expression across geographic localities.

## Results

### Sequencing, alignment, and transcript assembly

**Fig. 1:**
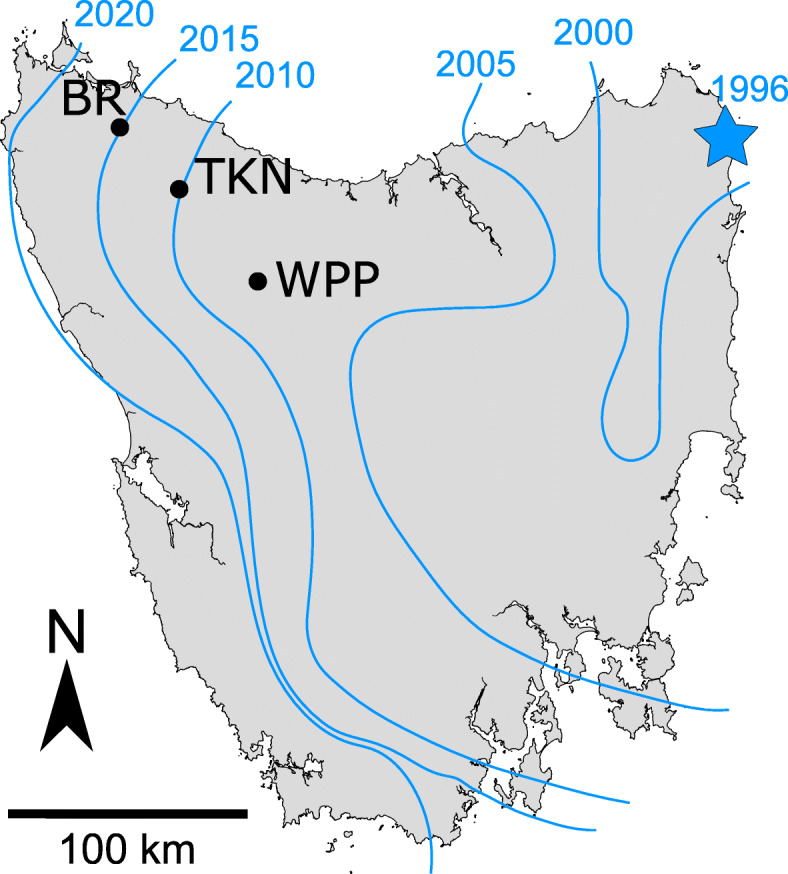
The east-west spread of DFTD since its origin in 1996 (approximate location indicated with blue star). Approximate location of the disease front over time is indicated as blue lines labelled by year. Study locations – West Pencil Pine (WPP), Takone (TKN), and Black River (BR) – are indicated. Lines depicting disease front adapted from [45]

To identify expression differentiation across hosts and tumor and test the four predictions above, we sequenced mRNA libraries from 58 tissue samples from 39 devils (20 males, 19 females). Samples were collected from wild devils between 2016 and 2018 from three distinct localities (Fig. 1): Black River (BR; first infected in 2016), Takone (TKN; first infected in 2011) and West Pencil Pine (WPP; first infected in 2006). Samples comprised lip biopsies from 20 devils putatively uninfected by DFTD, and paired lip and tumor biopsies from 19 devils (38 samples) clinically infected with DFTD. Volumes of sampled tumors did not significantly differ among localities (analysis of variance: *P* = 0.365) We generated a total of 1,168,476,356 sequence reads, which were reduced to 1,167,199,873 following quality trimming and filtering (per sample mean = 20,124,136; SD = 5,905,604). Reads aligned to the reference genome at an average of 91.9% per library (SD = 3.4%), with 63.1% (SD = 3.9%) of bases mapping to annotated mRNA regions.

### Differential gene expression

#### Between devil lip and DFTD tissues

**Fig. 2:**
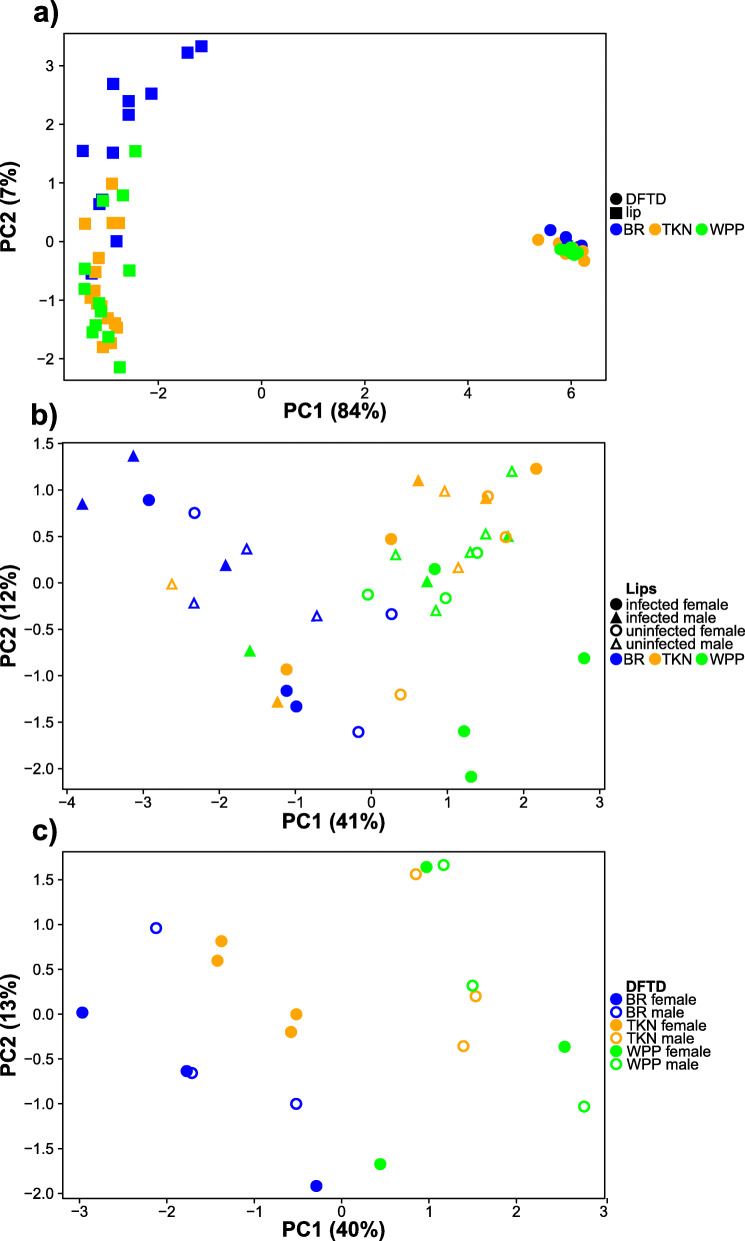
Variation in gene expression among a) DFTD tumor and Tasmanian devil lip biopsies, b) lip biopsies sampled from devils of different sex and DFTD infection status, and c) DFTD tumors sampled from devils of different sex and from different localities. Each plot was generated using the 500 genes exhibiting the most expression variation (i.e., the highest standard deviation across all samples) from each differential expression analysis

For the comparative analysis of differential gene expression between lip and tumor tissues, we retained a total of 14,807 expressed genes after filtering (Additional file 8: File S8). We found a dramatic difference in gene expression between lips and tumors (11,149 significant differentially expressed genes at a false discovery rate [FDR] of 0.05), which were clearly delineated as multidimensional scaling clusters (Fig. 2a; Additional file 5: Fig. S5). Because the number of differentially expressed genes for this contrast was so large, we applied a log2 fold change (log2FC) minimum threshold of ±2 to focus our efforts on only the most highly differentially expressed genes. With this more stringent filter, we identified 4234 genes that were still differentially expressed between tissue types, with 2286 upregulated and 1948 downregulated in DFTD tumors.

To evaluate variation among localities in genes differentially expressed between lip and tumor, we analyzed differential expression between lips and tumors for each locality separately and detected similarly high numbers of differentially expressed genes. We identified 4282 genes differentially expressed at log2FC ± > 2 in BR (646 DE genes unique to BR), 4238 in TKN (268 unique), and 4181 in WPP (350 unique).

Using the PANTHER Overrepresentation Test [46], we identified numerous biological processes that were significantly enriched among genes that were highly differentially expressed between tumor and lip tissues (Additional file 6: Fig. S6). Many of these gene ontological (GO) terms were clearly associated with cellular differentiation due to the epidermal and skeletal muscle tissue present in lips as opposed to the Schwann cell (a peripheral nerve cell) origin of DFTD. For example, genes upregulated in tumors were enriched for nervous system processes and extracellular matrix organization but depleted in DNA damage response, transcription, and protein metabolism. Genes downregulated in tumors were enriched for muscle cell development and function but depleted in double-strand break repair, negative cell cycle regulation, transcription, and translation. Of genes that were differentially expressed between lips and tumors that were unique to a given locality, only BR yielded significantly enriched biological processes. Overrepresented processes were associated with regulation of cell shape and signaling, whereas underrepresented processes were associated with gene expression and metabolism. Full lists of significantly over- or underrepresented GO-terms are provided in Additional file 9: File S9.

**Fig. 3:**
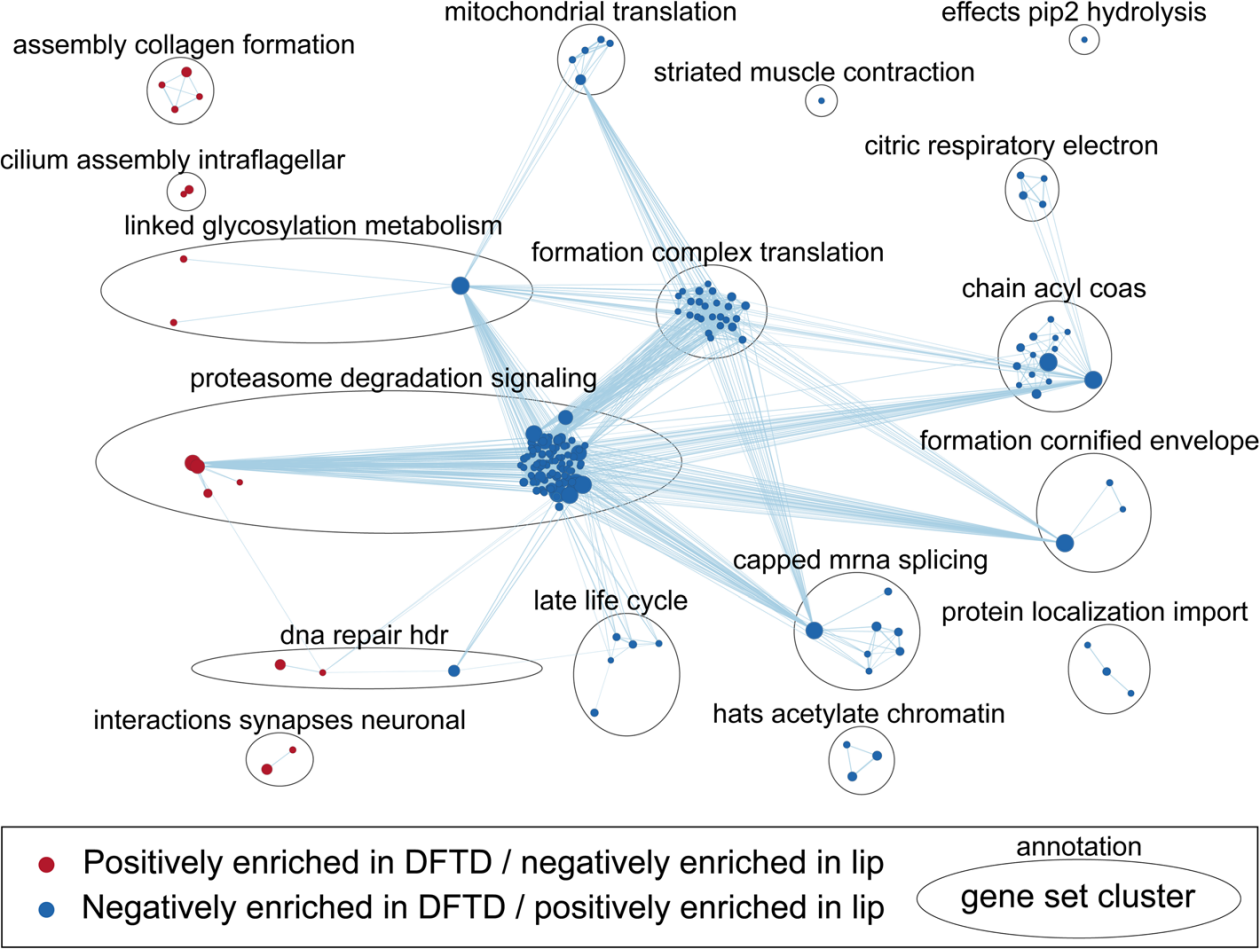
Enrichment Map showing result from gene set enrichment of DFTD tumor tissue compared to normal devil lip tissue. Nodes represent gene sets; red indicating significant positive enrichment in DFTD, and blue indicating significant negative enrichment in DFTD. Edges indicate sharing of genes between gene sets, with gene set clusters, delineated by ellipses, defined by large numbers of shared genes. Cluster annotations are derived from highly repeated words among gene set names

Gene Set Enrichment Analysis revealed 16 positively and 163 negatively enriched Reactome pathways in DFTD at FDR = 0.01 (Fig. 3). Summarization via EnrichmentMap clustering highlighted a variety of generalized differences between tumor and lip tissues. The largest pathway gene set cluster contained 95 pathways and was annotated “proteasome degradation signaling” (Fig. 3). This cluster contained only four pathways that were positively enriched in DFTD, which were associated with the mitotic cell cycle. The remaining pathways in this cluster were negatively enriched in DFTD, including many that were associated with the immune system and various signaling pathways such as Notch, slits and robos, and DNA damage checkpoints (both dependent and independent of P53). A closely related cluster annotated as “DNA repair HDR”, contained pathways associated with homology-directed DNA repair that were positively enriched in DFTD, as well as pathways negatively enriched in DFTD associated with transcriptional regulation by TP53 (Fig. 3). Clusters entirely positively enriched in DFTD were associated with collagen formation and extracellular matrix organization, cilium formation, and neuron function (Fig. 3; see Additional file 10: File S10 for full list of enriched gene sets and annotated gene set clusters).

#### Among devil lip tissues

For comparison among lip tissues only, we retained a total of 14,165 expressed genes (Additional file 8: File S8). Overall, we found little evidence of differential gene expression between lips from devils of different sex or based on infection status, irrespective of sampling locality. No genes were differentially expressed in any of our contrasts between DFTD-infected devils and those from clinically healthy devils, irrespective of host sex (Fig. 2b). However, when contrasting host sexes directly regardless of infection status, we identified seven differentially expressed genes. Specifically, we observed significant upregulation of *FRMD7*, *HMGB3*, *MECP2*, and three uncharacterized novel genes in females, and upregulation of one uncharacterized novel gene in males (see Additional file 13: Table S13 for full names and descriptions of gene symbols). No additional genes differentially expressed between males and females were identified when considering DFTD-infected and uninfected devils separately. Both groups exhibited significant upregulation in females of *FRMD7*, *HMGB3*, and two uncharacterized novel genes, whereas *MECP2* was upregulated in females only among uninfected individuals. Among localities, we identified 1564 genes differentially expressed between TKN and BR (897 up and 667 downregulated in TKN) but no evidence of differential expression in lips between any other pair of localities. Genes differentially expressed between TKN and BR were associated with immune system processes and cellular developmental processes (upregulated in BR), as well as lipid metabolism (upregulated in TKN) (Additional file 9: File S9). Six gene sets were positively enriched in BR relative to TKN and were associated with phagocytosis and T-cell activation (Additional file 10: File S10). For all other lip-only contrasts, there were insufficient numbers of differentially expressed genes for GO-term enrichment analysis. Similarly, no significantly enriched pathway gene sets (FDR = 0.12–1.0) were identified for any other lip-only statistical contrasts.

#### Among tumor tissues

In DFTD tumors, we retained 14,204 expressed genes following filtering (Additional file 8: File S8). Genes expressed in DFTD but not lip tissues, and vice-versa, were almost entirely linked to functions associated with the cell type of origin (i.e., Schwann vs muscular/epidermal cells). When contrasting tumors infecting devils of opposite sex, we found no genes that were significantly differentially expressed between sexes across all localities, but we observed some locality-specific differences. Although no genes were differentially expressed between tumors from different host sexes in BR, 17 genes were differentially expressed in tumors between sexes in TKN, all of which were upregulated in tumors from male devils relative to females: *SYNE2*, *CLUH*, *PARS2*, *VPS13A*, *KIAA1586*, *TASOR2*, *SLC12A5*, *TRRAP*, *DIP2A*, *BRPF1*, *PCNX3*, and six uncharacterized novel genes. In WPP, only *MRPL53* was differentially expressed – upregulated in tumors infecting female devils.

Direct contrasts between localities – irrespective of host sex – revealed substantial differences in tumor gene expression. In general, the greatest difference was between BR and WPP, with TKN being somewhat intermediate of the two other localities (Fig. 2c, Additional file 7: Fig. S7). In all, 3825 genes were differentially expressed between BR and WPP, of which 1635 were upregulated and 2190 were downregulated in BR compared to WPP. In BR tumors relative to TKN, 414 genes were upregulated, and 548 genes were downregulated. In WPP tumors relative to TKN, 823 genes were upregulated, and 416 genes were downregulated.

All three pairwise contrasts of tumors between localities were significantly enriched for biological processes. All processes overrepresented among genes upregulated in BR relative to TKN were also overrepresented among genes upregulated in BR relative to WPP – these were broadly associated with translation and protein synthesis. Additional processes overrepresented among genes upregulated in BR relative to WPP were predominantly associated with other metabolic and biosynthetic processes. Genes downregulated in BR relative to WPP were disproportionately associated with chromosome organization and gene expression. Similarly, in TKN tumors relative to WPP, downregulated genes were also associated with chromosome organization and gene expression regulation, whereas upregulated genes were associated with transcription, translation, and various metabolic and biosynthetic processes.

**Fig. 4:**
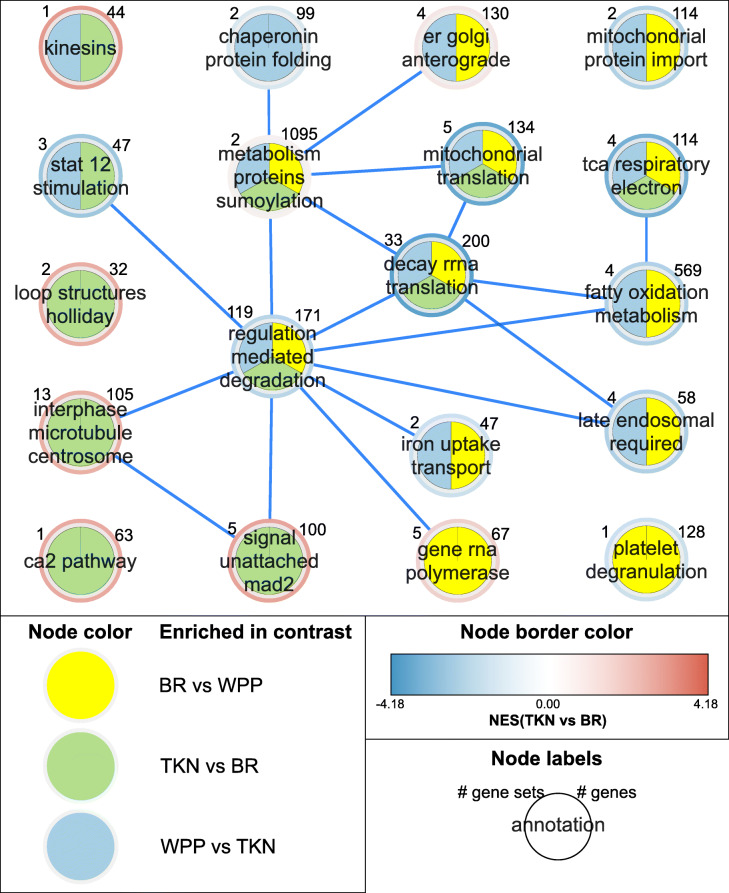
Three-way enrichment Map showing gene set clusters (nodes) that were significantly enriched in DFTD tumors between pairs of localities. Node colors indicate which between-locality contrasts were enriched for gene sets within each cluster (e.g., clusters showing all three colors contain gene sets that were enriched in all three pairwise contrasts between localities). Node border colors indicate average normalized enrichment scores (NES) for gene sets in TKN tumors relative to BR tumors. Edges represent genes shared between gene sets from different clusters. Clusters are annotated and labelled according to the number of genes and gene sets they contain. Cluster annotations are derived from highly repeated words among gene set names

We identified significantly enriched Reactome pathway gene sets in comparisons between DFTD tumors sampled from different host sexes in TKN, as well as in comparisons among tumors from different localities generally. The most pronounced difference (in terms of both the number of enriched pathways as well as the magnitude of enrichment) was between BR and WPP, with TKN intermediate of the two but generally more similar to BR. Interestingly, the pathway gene set clusters positively enriched among tumors from WPP compared to other localities were characteristic of those positively enriched in DFTD compared to devil lip tissue. We identified five clusters of pathway gene sets that were significantly enriched in all three pairwise contrasts between localities (Fig. 4). The largest of these clusters – “regulation mediated degradation” – contained 119 pathways and was approximately equivalent to the largest cluster identified in the DFTD-lip contrast above. Accordingly, it contained pathways predominantly associated with cell cycle checkpoints and programmed cell death, and the innate and adaptive immune system; these pathways were all significantly negatively enriched in WPP tumors compared to those from TKN and BR. A small number of pathways in this cluster associated with the mitotic cell cycle were positively enriched in WPP compared to other localities (Fig. 4; see Additional file 10: File S10 for full list of enriched gene sets and annotated gene set clusters).

Of the three between-locality tumor contrasts, the TKN-BR contrast appeared to be the most distinct, with four pathway gene set clusters that were significantly enriched only in TKN-BR, while eight clusters were enriched in the other two between-locality contrasts but were not enriched in TKN-BR (Fig. 4). Clusters unique to the TKN-BR contrast included many pathways associated with the mitotic cell cycle (Fig. 4). Clusters that were lacking only from the TKN-BR contrast included pathways associated with protein folding and transport, metabolism, negative regulation of transcription, and autophagy (Fig. 4).

In general, we observed no enrichment of pathways among tumors sampled from devils of different sexes, either across all localities or within localities individually, with the exception of TKN. As reflected in Fig. 2c, gene set enrichment between tumors sampled from different sexes from TKN generally mirrored enrichment between WPP and BR, with TKN males negatively enriched for pathways that were also negatively enriched in WPP when compared to BR. Overall, at FDR = 0.05, there were four pathways significantly positively enriched and 21 negatively enriched in TKN males compared to females. Pathways positively enriched in TKN males were associated with chromatin organization and mitotic prometaphase. Pathways negatively enriched in TKN males were associated with eukaryotic and mitochondrial translation and protein synthesis, nonsense-mediated decay, and respiratory electron transport.

### Genotypic variation and associations with gene expression

**Fig. 5:**
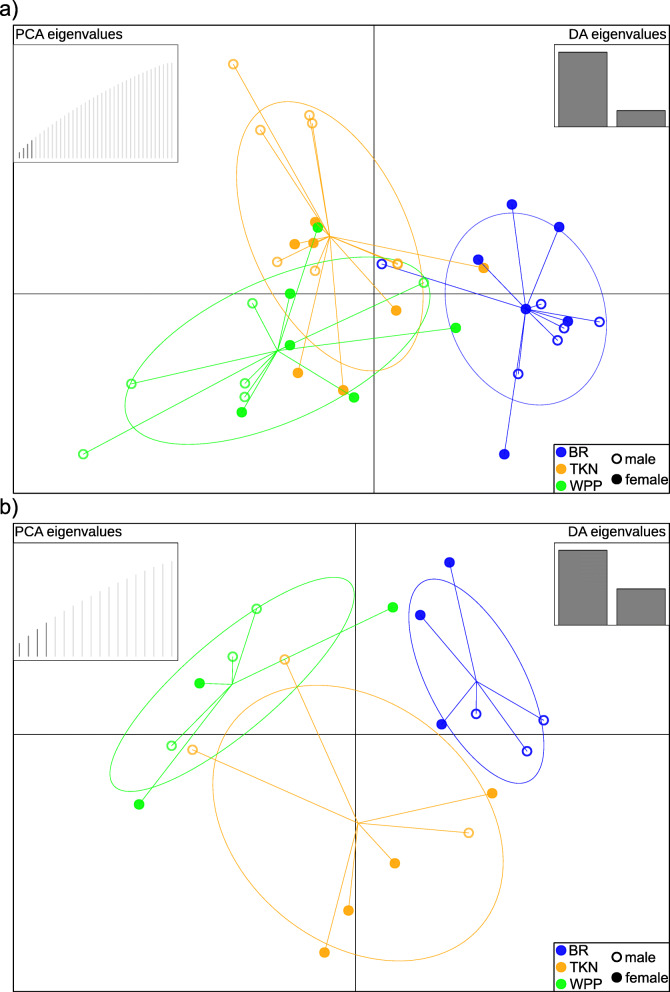
Discriminant analyses of principal components, showing weak genetic structure in a) Tasmanian devils and b) devil facial tumor disease from West Pencil Pine (WPP), Takone (TKN), and Black River (BR)

To identify genotypic variation potentially associated with variation in gene expression, we genotyped indels and single nucleotide polymorphisms (SNPs) from the transcriptome sequences. Following filtering (see Additional file 3: Text S3), we retained 9559 SNPs and 6013 indels in devils, and 473 SNPs and 1554 indels in tumors (including 100 alleles private to BR, 121 to TKN, and 112 to WPP) for analysis. For both tissue types, we found evidence for weak genetic structure when specifying sample localities a priori, though this structure was more pronounced in devils than in tumors (Fig. 5). However, when identifying genetic clusters purely from SNP variation, only one cluster each was supported for both devils and tumors.

We used SnpEff [47] to annotate all genotyped tumor variants according to their positions with respect to known genes and each variant’s predicted impact on gene function. 4203 variant-gene effects were predicted in tumors, comprising 2264 variants associated with 1248 genes (note: loci may contain multiple variants, and variants may have multiple effects involving different genes). Of these variant-gene effects, 1514 involved variants located in intergenic regions, likely due to sequencing of noncoding mRNA, unannotated exons, or, potentially, contamination by genomic DNA. By comparison, similar proportions of intergenic variants have been detected in other RNA-seq studies [e.g., 48, 49]. Most other variants were also in non-coding regions: 159 were < 5 kb upstream, 20 were located in the 5′ UTR, 211 were intronic, 392 were located in the 3′ UTR, and 1617 were < 5 kb downstream. Only 290 variants were in exons and were not significantly enriched for any biological process. Of these exonic variants, 77 were identified as missense variants, seven were nonsense variants, and none were nonstop variants.

For 4088 tumor genes that were differentially expressed between any pair of localities, 444 contained variants with predicted effects on gene function. By comparison, 920 of 10,116 non-differentially expressed genes also contained variants with predicted effects, suggesting that variant-gene effects were equally likely to be detected within differentially expressed compared to non-differentially expressed genes. Following filtering for biallelic variants, we retained a total of 413 gene-expression-locus combinations for Kruskal-Wallis tests. Including loci with multiple predicted effects, these comprised 238 intergenic, 27 upstream, three 5′ UTR, 47 intronic, 88 3′ UTR, 357 downstream, and 89 exonic variants. However, after FDR adjustment, we found no significant associations between any variant on the expression of the genes it was predicted by SnpEff to effect.

## Discussion

As predicted, substantial differentiation exists in gene expression between lip and tumor tissues. We observed differential expression patterns consistent with DFTD’s origin as a Schwann cell tumor [22], including genes involved in nervous system functions such as synaptic signaling and ion transport, as well as characteristics common to cancers [5]. Lips, in contrast, had differentially expressed genes characteristic of muscle and epidermal tissue function. Overall transcriptional variation appeared to be higher among lip tissues compared to that observed among tumor tissues, potentially due to greater genetic variation among devils or exposure of lip tissues to greater environmental variation. However, in lip tissues, we found surprisingly little transcriptional variation associated with sex or infection status, despite clear sex-biased differences in resistance and tolerance of DFTD [34, 39]. Perhaps most interestingly, we found significant variation in gene expression among DFTD tumors collected from different localities, potentially associated with different tumor strains, environmental effects (either within-host or extrahost) or host-pathogen coevolutionary relationships. However, we found no association between detected SNP variants in transcribed genomic regions and gene expression, consistent with results from previous studies that showed variants in regulatory regions [40], which are not captured in RNA-seq approaches, likely affect observed phenotypic variation.

### Gene expression characteristics of DFTD

Despite considerable genotypic and phenotypic diversity among cancers [50], there are relatively common mutational drivers that are necessary for the genesis and proliferation of cancerous cells. Functions of these drivers include: the sustenance of proliferative signaling, evasion of growth suppressors, resistance to apoptosis, induction of angiogenesis, activation of invasion and metastasis, reprogramming of energy metabolism, and evasion of immune response [51, 52]. Similarly, we observed differential expression of genes and enrichment of pathways in DFTD associated with the extracellular matrix, the cell cycle, DNA replication and repair, and immune function. The extracellular matrix is fundamental to maintaining tissue homeostasis. Dysregulation of the extracellular matrix can both cause – and occur as – a response to the development and proliferation of cancerous cells, facilitating uncontrolled proliferation, angiogenesis, tissue migration and invasion, and metastasis [53–55]. Cell cycle ‘checkpoints’ detect DNA damage or errors in DNA replication and chromosome organization and are regulated by critical tumor suppressor genes that prevent proliferation of defective and potentially cancerous cells [56]. A key example of such a gene is *TP53*, which is activated in response to DNA damage, initiates the inhibition of cell proliferation, and regulates apoptosis [57]. Loss of *TP53* function is common to many cancers [57, 58]. We found various pathways associated with *TP53*, such as G1 and G2 cell cycle checkpoints, to be downregulated in DFTD relative to normal lip tissue. In turn, upregulation of pathways associated with mitosis and the cell cycle that likely lead to increased cell proliferation were also found in DFTD.

Despite downregulation of cell cycle checkpoint and apoptosis pathways, we observed upregulation of homology-directed DNA repair (HDR) in DFTD. Dysfunctional HDR often results in genomic instability and can lead to carcinogenesis [59, 60]; however, cancers can become resistant to standard cancer treatments aimed at inducing DNA damage through reactivation of HDR, emphasizing that cancer cell proliferation is not dependent on HDR suppression [61]. Active yet dysfunctional HDR can lead to errors in double-stranded break repair that produce gross chromosomal rearrangements [62], such as those that are evident in DFTD [21, 24, 63].

Although all cancers exhibit some form of immune avoidance, DFTD and other transmissible tumors are unique in that they can evade host MHC recognition of non-self-cells [27]. In DFTD, MHC avoidance is achieved through the ERBB-STAT3 axis [44]. Specifically, DFTD likely became transmissible when a normal Schwann cell tumor gained a variant exhibiting overexpression of ERBB3, which overactivates the transcription factor STAT3 and blocks the production of MHC I [26, 27, 44]. Without MHC I expression, DFTD cells are unable to be recognized as foreign by the host. Similar to previous work, we found the differential expression of other genes associated with STAT3, including upregulation of *MMP2*, *HDAC5*, and downregulation of *PTGIS* [44]. However, in contrast to this previous study that found upregulation of the TRIM28 protein, which is typically activated in response to STAT3 signaling, we detected slight downregulation in expression of this gene. *TRIM28* is often overexpressed in cancer [64]; however, its expression has been shown to be predictive of tumor class in human glioblastomas [65], and positively correlated with tumor size and development stage, whilst being negatively correlated with patient survival in human hepatocellular carcinoma [66]. Lack of detectable *TRIM28* overexpression may thus reflect size or developmental stage variation among the sampled DFTD tumors.

We also observed downregulation of Notch signaling, which is critical to Schwann cell development and required for differentiation of Schwann cell precursors (SCP). Notch activation upregulates *ERBB2*, which acts as a receptor for *NRG1*, which in turn is critical for transitioning SCPs to immature Schwann cells [67, 68]. In the absence of Notch signaling, a scarcity of ERBB2 receptors would lead to reduced sensitivity of SCPs to *NRG1*, despite its overexpression. Dysfunction of these pathways suggests a decoupling of DFTD cell proliferation from typical Schwann cell developmental controls.

### Gene expression in devil lip tissue is not associated with infection status or sex

DFTD infection has no effect on gene expression in devil lip tissue. This is surprising as DFTD has significant effects on host physiology and can elicit host immune response [69]. Tumor growth leads to increasing metabolic demands concurrent with difficulty feeding, ulcerations, metastases, and secondary infections that produce a progressive loss of body condition and almost universal mortality within 12 months following visible tumor development [18, 30, 34]. In humans, gene expression in normal tissues adjacent to tumors reflects a state that is intermediate between healthy and cancerous, with commonly expressed pathways including pro-inflammatory responses induced by the tumor [70]. We chose lip tissue due to its proximity to the mouth, where DFTD allografts typically implant, believing that changes in gene expression would be greatest in tissues local to tumors. However, DFTD tumors do not always occur on the lips or in the mouth, and there was likely variation in the proximity of lip biopsies to the site of tumor growth. Ethical and experimental concerns necessitated a consistent sampling strategy for healthy tissues, preventing individual adjustments of biopsy locations to accommodate variation in tumor location. In addition, lip tissues may have been inappropriate for detecting systemic immunological or metabolic changes. For example, systemic immune responses associated with tumor growth tend to involve the accumulation of immune cells in the peripheral blood or lymphoid tissues, rather than in the epidermis [71]. Biological functions associated with immune response were not underrepresented in lip tissues, suggesting that the lack of differential expression was not specifically due to a lack of immune expression in these tissues.

Despite a lack of a direct immune response in devil lips to DFTD infection, we observed upregulation of immune-associated genes in BR relative to TKN devils, irrespective of infection status. This difference is not necessarily associated with DFTD and may be due to differential exposure to other infectious agents or innate differences in immune function between genetically distinct populations. However, DFTD is an overwhelmingly strong selective force in devils [37, 39, 72] and likely drives immune adaptation in affected populations. Further, documented immune responses to DFTD [69] suggest that differential host immune expression between localities may alter the microenvironment faced by invading tumor cells. We thus recommend further gene expression studies targeting immunologically active host cells; specifically, to investigate systemic host responses to DFTD using blood samples as well as a refined approach for detecting localized responses by targeting healthy tissues < 1 cm from DFTD tumors.

Male and female devils exhibit different responses to DFTD, with females losing body condition at a significantly slower rate when infected and genetic evidence of adaptations that result in greater survival rates among females [34, 39]. Although DFTD infection produced no transcriptomic response in lip tissues for either sex, we found several genes that were differentially expressed between males and females generally, regardless of infection status. Consistent with previous work comparing sexes in uninfected devils [73], the X-linked gene *FRMD7* was downregulated in males (log2FC = − 4.01). *FRMD7* is putatively involved in fatty acid metabolism and has been associated with skin disorders, serving as a potential factor in differential susceptibility between sexes to DFTD [73]. Additionally, we found six other genes that were differentially expressed between sexes. Four of these were uncharacterized, while *HMGB3* and *MECP2* – both also X-linked – were downregulated in males. HMGB3 is a DNA-binding protein that helps maintain stem cell populations and is overexpressed in some human cancers via the Wnt signaling pathway [74, 75]. Further, HMGB3 affects nucleic acid recognition and innate immune system activation, and its upregulation has been associated with allograft rejection [76–79]. Therefore, higher baseline expression of *HMGB3* in female devils may enhance the innate immune response to DFTD relative to males. Recently, *MECP2* was identified as an oncogene through induction of the MAPK and PI3K growth factor signaling pathways and is overexpressed in many human cancers [80]. However, it is unclear how *MECP2* expression in normal host tissues could affect DFTD progression.

### DFTD tumor gene expression varies geographically

We observed considerable variation in tumor gene expression among the sampled localities, which varied in the length of time since DFTD arrival. Interestingly, gene expression patterns that we observed in DFTD relative to host lips were more intense (i.e., more differentially expressed genes and greater log2 fold-changes) in WPP tumors – where DFTD has been present longest among our study sites – than in other populations. Specifically, WPP tumors exhibited upregulation of mitosis and downregulation of translation, DNA damage checkpoints, and immune function relative to tumors from BR, which had the shortest time since DFTD arrival. Varying intensity of DFTD-characteristic gene expression may be due to differences in the ratios of normal-to-tumor cells within tumor biopsies, potentially driven by subtle differences in tumor morphology among localities (e.g., differences in the extent to which tumor tissue is delineated from the surrounding host tissue, or the distinctness of tumor margins). On the other hand, expression changes in cancer-associated genes are not only linked to tumorigenesis but can be directly correlated with tumor aggressiveness and overall prognoses in human cancer patients [81, 82]. Meanwhile, incrementally greater gene expression changes through time can produce progressively more severe phenotypes or reflect more advanced stages of tumor development [e.g., 83, 84]. The strength of expression in DFTD-associated pathways therefore may be associated with DFTD phenotypic variation, such as growth rates, although no such differences (nor differences in tumor morphology) between the studied localities have been documented.

Gene expression in tumors from TKN reflected an intermediate state between WPP and BR but were differentiated by sex (Fig. 2c; Additional file 7: Fig. S7). That is, tumors from TKN males exhibited patterns of gene expression that were characteristic of WPP tumors from both sexes, and tumors from TKN females resembled male and female tumors from BR (see Fig. 2c). Perhaps coincidentally, previous work demonstrating more rapid body condition loss in DFTD-infected males than in females was conducted in TKN [34], while no similar analysis of host body condition has been performed for either of our other study sites. We do not have sufficient serial volume measurements for the tumors in our study to directly associate gene expression with tumor growth rates. In the absence of data comparing tumor growth rates or host body condition between TKN, WPP, and BR, it is difficult to establish a link between relative levels of DFTD-characteristic gene expression and the severity of disease.

Although it remains unclear whether transcriptional variation in DFTD occurs among localities as a result of neutral or adaptive differences among strains, there is an interesting temporal trend that correlates with expression changes. DFTD first arrived in WPP in 2006 and has thus had the longest amount of time to adapt to devils in that locality. DFTD then emerged in TKN in 2011, and tumors there showed intermediate expression values between WPP and BR, where DFTD has been for the shortest time (since 2016). Thus, expression variation among study sites may reflect ongoing DFTD adaptation to devils within populations, which themselves have demonstrated rapid evolutionary responses to DFTD [37, 39]. Additionally, multiple DFTD lineages with differing degrees of pathogenicity have been observed. Specifically, in WPP, DFTD arrival in 2006 was characterized by initially low mortality rates compared to localities that had experienced DFTD for longer [85]. Karyotype analysis confirmed the existence of a distinct tetraploid strain at WPP that was replaced by a more virulent diploid strain in 2012, causing an immediate increase in disease prevalence and population decline [86]. In addition, recent phylodynamic analysis indicates multiple tumor lineages exhibiting differences in transmission rates, demonstrating epidemiological variation among distinct tumor strains [87]. Given its recent emergence, our BR tumor samples may represent a novel DFTD lineage present near the advancing disease front. Such a lineage was not observed at a broad spatiotemporal scale [87] but more intensive sampling of the most recently emerged tumors near and on the west coast of Tasmania provides evidence of spatially structured tumor lineages in this region [88]. Specifically, WPP contained tumors from multiple distinct lineages, whereas tumors sampled from further west (closer to the vicinity of our TKN and BR sites) almost entirely comprised a single lineage [88]. Spatial structuring of tumor lineages may therefore explain the observed transcriptional variation among localities.

We detected weak genetic structure in tumors that broadly reflected transcriptomic variation among localities and may reflect different tumor strains. However, we acknowledge that the persistence of a small number of host variants in the tumor dataset may produce a residual signal of host population structure, despite rigorous bioinformatic filtering to exclude known devil variants genotyped from lip RNAseq data as well high-coverage whole genome sequences (see Additional file 3: Text S3). Further, although we identified a number of variants in tumors that were predicted to affect the function of genes differentially expressed among localities, no significant associations were detected. Thus, transcriptional variation in tumors may be purely regulatory in origin, or we may have lacked sufficient statistical power (19 samples) to identify genotype-expression associations. Alternatively, population genetic structure and adaptive and plastic responses to the local environment in hosts can produce variation in immune function that drive differential responses to wildlife disease [14, 89]. Our study sites have similar vegetation communities and experience similar climatic conditions but decrease in elevation from east to west. Region-specific adaptation of devils to local environmental conditions exists, as well as significant selection imposed by DFTD following its arrival in naïve populations [37, 39, 72]. Different transcriptomic responses of DFTD to devils from different localities may thus also be explained by immunological variation among devil populations that is driven by environmental differences that lead to differential exposures to non-DFTD infectious agents.

## Conclusions

Characterization of DFTD gene expression and our broad analysis of transcriptomic variation in devils and DFTD points to a potential link between the relative degrees of DFTD-characteristic gene expression patterns and host population. Whether this variation is due to distinct tumor strains with differing phenotypes or by locally specific differences in host tolerance/resistance remains unclear. Nonetheless, geographic variation in gene expression among tumors highlights the potential for ongoing devil-tumor evolution. DFTD arrived at each of the populations in this study at different times, and thus the coevolutionary processes that influence the nature of host-tumor molecular interactions may be at different stages for each. Alternatively, or perhaps concurrently, environmental variation among localities may also be influencing spatial variation in tumor gene expression. To separate these alternative explanations would require replicated sampling of time points with respect to DFTD arrival across different localities. Given the well-documented east-to-west spread of DFTD over a 25-year period, this system is highly suited to exploration of host-pathogen coevolutionary relationships at various times since disease arrival.

## Methods

### Sample collection

Lip and tumor tissues were collected as 3 mm biopsies from live, wild Tasmanian devils between 2016 and 2018 from three locations in northern Tasmania, Australia: Black River (BR), Takone (TKN), and West Pencil Pine (WPP) (Fig. 1; Additional file 1: Table S1). Detailed descriptions of ethically-approved trapping protocols can be found in Hawkins et al. [19] and Hamede et al. [86]. All research involving use of animals and field activity was performed under University of Tasmania ethics approval A13326 and WSU IACUC approval ASAF 6796. Normal (i.e., noncancerous) tissue from inside of the lip was chosen due to its proximity to where DFTD tumor allografts typically implant, increasing the likelihood of detecting localized tissue responses in DFTD-infected animals, while ensuring both animal and handler safety. Where individuals had multiple tumors, biopsies were taken from the largest tumor. All tumor biopsies were taken from ulcerated tumors and comprised only solid tissue that was free from necrosis or secondary infection. Sampled tumors were measured, and tumor volumes were calculated using the ellipsoid volume equation ($volume=\frac{4}{3}\times \pi \times \frac{length}{2}\times \frac{width}{2}\times \frac{depth}{2}$). Among-population differences in tumor volume were evaluated using an analysis of variance. For each population, lip biopsies from at least 3 DFTD-infected and 3 uninfected devils of each sex were collected, along with corresponding tumor biopsies from every infected devil. All biopsies were immediately preserved in RNAlater, kept at − 20 °C for up to two weeks while in the field, and subsequently stored at − 80 °C until RNA extraction.

### RNA extraction, library preparation, and sequencing

We extracted whole RNA from lip and tumor samples in two batches. The first batch contained samples collected in 2016, and RNA extraction was performed using a combination of Nucleospin RNA extraction kit (Macherey-Nagel, Easton, PA, USA), Qiagen Allprep DNA/RNA Mini Kit (Qiagen Inc., Hilden, Germany) and a standard Trizol-chloroform protocol. The second batch contained samples collected in 2018, and RNA extraction for this batch was performed using the Trizol-chloroform protocol. All extracted RNA was treated with DNAse prior to library preparation to remove DNA contamination.

We prepared and sequenced mRNA-seq libraries in three batches. Batches 1 (*n* = 4) and 2 (*n* = 12) comprised samples collected in 2016; we prepared these libraries using the NEBNext Poly(A) mRNA Magnetic Isolation Module (New England BioLabs, Ipswich, MA, USA) according to Fraik et al. [73]. Batch 3 (*n* = 42) comprised all samples collected in 2018, with library preparation performed by the Washington State University Genomics Core in Spokane using the Illumina TruSeq Stranded mRNA Library Prep Kit and assessed for quality using a Fragment Analyzer (Agilent, Santa Clara, CA, USA). Both library preparation kits employ a PolyA tail selection approach that isolates mRNA from ribosomal and globin RNAs. For each batch, samples were pooled together and sequenced for 100 bp paired-end reads using an Illumina HiSeq 2500: batch 1 on a single lane, batch 2 across two lanes, and batch three across four lanes. All sequencing was performed by the Washington State University Genomics Core, Spokane, WA. We accounted for potential batch effects attributable to use of different kits and sequencing lane effects throughout all analyses (see below).

### Sequence alignment and transcript assembly

We conducted initial quality screening of raw sequencing reads using FastQC and assessed study-wide sequencing quality using MultiQC. To trim and filter reads for quality, we used the TrimGalore wrapper with relatively relaxed settings due to the documented negative effects of stringent filtering on RNAseq analyses [90]. Specifically, we trimmed adapter sequences and ends with a Phred quality score < 10 and removed reads < 30 bp long.

We performed sequence alignments and assembled transcripts according to protocols recommended by Pertea et al. [91]. Using Hisat2 v 2.1.0 [92], we aligned the trimmed reads to the published Tasmanian devil reference genome (Murchison 2012), specifying the --dta flag to produce alignments suitable for transcript assembly. Aligned reads were checked for DNA contamination, firstly by visualization using the Broad Institute’s (Cambridge, MA, USA) Integrative Genomics Viewer, and then by using the DepthOfCoverage function from the Genome Analysis Toolkit (GATK) v 4.1.8.1 to quantify the proportions of different genomic regions (e.g., exonic, intergenic) that were covered by at least one sequencing read (see Additional file 12, Table S12 for results). We used samtools to sort the alignments prior to assembling the transcripts using Stringtie v 2.1.0 [93] and the Ensembl reference annotation v 7.0.97 [94]. Then, using Stringtie, we merged all transcripts into a non-redundant assembly and used gffcompare v 0.11.7 [95] to annotate the merged assembly and examine how the assembled transcripts compare with the reference annotation. We then repeated the transcript assembly for each sample, using the merged annotation as the reference, and generated tables of transcript abundances including only transcripts that appeared in the merged reference annotation.

### Differential expression analysis

Prior to differential expression analysis, tables of transcript abundances were converted into read tables of gene-level abundances using the R package tximport [96]. All differential expression analyses and preparatory steps were conducted in R using EdgeR [97] unless otherwise indicated, with the entire pipeline run three times as distinct analysis sets: 1) tumor-lip contrasts to investigate differential expression in tumors compared to host lip tissues; 2) lip-only contrasts to investigate variation in gene expression among hosts (e.g., with respect to sex and/or DFTD infection status); 3) tumor-only contrasts to investigate variation in expression among tumors (e.g., with respect to host sex or locality). For the tumor-lip contrasts, all lip and tumor transcriptomes were analyzed together, while the lip-only contrasts only included lip transcriptomes and the tumor-only contrasts only included tumor transcriptomes.

To ensure our analysis contained only genes that were expressed across experimental groups, we removed genes lacking transcript counts > 10 in at least three samples. Read counts were then normalized among libraries using the *calcNormFactors* function. We included the following factors in our analysis: *sex* (of the host devil, in the case of tumors)*, infected* (i.e., with or without DFTD), *tissue* (normal lip or DFTD tumor), *population* (sampling locality; BR, TKN, or WPP), and *batch* (batches 1 or 2 comprising 2016 samples, or batch 3 comprising 2018 samples). Differential expression analyses were performed using a quasilikelihood generalized linear model framework [98], using a different model formula depending on the tissues being analyzed. To simplify the design for each model and more easily facilitate comparisons between groups of potentially interacting factors, two factors of interest were combined into a single *group* factor. For the lip-tumor and lip-only models, this *group* factor comprised *sex* and *infected* (female-uninfected, female-infected, male-uninfected, or male-infected). For the tumor-only model, the *group* factor comprised *sex* and *population* (female-BR, female-TKN, female-WPP, male-BR, male-TKN, or male-WPP). For all models, to account for any effect that differences in lab protocols (see above) may have had on gene expression measurements, we included *batch* as a factor. Accordingly, we specified the formula for the host lip tissue vs DFTD tumor tissue model as:

$$ 0\sim tissue+ group+ population+ batch $$

The model formula for comparisons among lip tissues only was:

$$ 0\sim group+ population+ batch $$

The model formula for comparisons among tumor tissues only was:

$$ 0\sim group+ batch $$

For each model, we calculated log gene counts per million (logCPM) and constructed multidimensional scaling (MDS) plots using the R package limma [99] to identify clustering of samples according to our factors of interest. We observed MDS clustering of samples with respect to sequencing batch; this effect was subsequently corrected in all data visualization using the limma function *removeBatchEffect* to remove any variation in logCPM associated with the *batch* factor (Additional file 4: Fig. S4). MDS plots represented the top 500 differentially expressed genes defined as those with the largest standard deviations between all samples.

To identify differentially expressed genes between lip and tumor tissues, we performed a single contrast comparing all lip samples with all tumor samples, together with three separate contrasts comparing lip samples with tumor samples within in each locality. For the tissue-specific models, we performed a number of different contrasts: among lip samples, we performed 10 contrasts comprising various combinations of sex and infection status (Additional file 2: Table S2); among tumors, we performed seven contrasts comprising various combinations of host sex and sampling locality (Additional file 2: Table S2). Each contrast produced a table of results for all expressed genes quantifying the magnitude of differential expression between groups (log2 fold change; log2FC) and the significance of this difference given as nominal and false-discovery-rate-adjusted [FDR; 100] *P* values. We defined significantly differentially expressed genes at a false discovery rate threshold of 0.05, with no cutoff for log2FC. To verify effective control of batch effects, all differential expression results were compared with repeated analysis of batch 3 samples only (Additional file 11: File S11).

### Enrichment analysis

For contrasts with large numbers of differentially expressed genes, we performed a PANTHER Overrepresentation Test to test for biological processes (as defined by the PANTHER GO-Slim Biological Process annotation dataset) that are overrepresented among sets of differentially expressed genes. This comprises a Fisher’s Exact Test for GO-terms that are over- or underrepresented relative to a ‘background’ reference list, which we specified as the full list of expressed genes from which the differentially expressed genes were detected. Significantly enriched GO-terms were identified at an FDR threshold of 0.05. We performed overrepresentation tests on sets of significantly over- and under-expressed genes separately as this has been shown to be more powerful for detecting differentially expressed pathways than analyzing all differentially expressed genes together [101]. To reduce redundancy among significantly enriched GO-terms and thus reduce the size and increase interpretability of our GO-term lists, we used REVIGO [102] to cluster highly related GO-terms together and identify single representative GO-terms for each cluster.

To test for general patterns of up- or downregulation of biological pathways, we performed Gene Set Enrichment Analyses [GSEA; 103, 104] using pre-ranked lists of genes from differential expression analyses. Each expressed gene, irrespective of whether it was significantly differentially expressed, was ranked using a metric that measures the magnitude of the gene’s log2FC as well as the nominal statistical significance of that change, calculated as *log*2*FC* ∗  −  *log* 10(*P*). This produced a list whereby, for a given comparison between conditions, significantly upregulated genes had higher positive scores, significantly downregulated genes had lower negative scores, and genes that were neither up- nor downregulated had scores close to zero. Given such a ranked list, GSEA tests for known sets of related genes that have disproportionately higher or lower rankings, calculating an enrichment score (ES) and FDR-adjusted *p*-value for each gene set tested. We performed the analysis using GSEA v4.0.3 [103, 104], testing gene sets from the most recent Reactome pathways database [105]. All GSEA contrasts were performed across 1000 permutations and restricted to gene sets containing between 15 and 5000 genes, with the ‘meandiv’ normalization mode selected. For contrasts with relatively few (< 50) differentially expressed genes, we used the weighted enrichment statistic, which places greater emphasis on genes at the top and bottom of the ranked list. For contrasts with larger numbers of differentially expressed genes (thus having important genes ranked further down the list), we used the classic enrichment statistic, which weights rank positions equally.

To visualize and aid interpretation of our GSEA results, we produced similarity networks that cluster enriched gene sets according to numbers of overlapping genes and annotated each cluster of gene sets by common functions using the *EnrichmentMap* [106] and *AutoAnnotate* [107] add-ons to Cytoscape v 3.8.0 [108]. This accounts for redundancy between gene sets, reduces the overall complexity of the GSEA results, and facilitates identification of both major functional themes as well as unique or distinct pathways from among potentially hundreds of significant yet largely redundant gene sets. Gene sets were filtered to exclude those above an FDR q-value cutoff of 0.01. Gene set clusters were defined using the Markov Cluster (MCL) algorithm with default settings. For each cluster, the top three most frequently occurring words among gene set names – normalized according to their frequency across all gene sets – were used to concisely annotate each cluster for generalized functions. We made minor manual edits to annotations where automatic annotation produced misleading results.

### Genotype analysis

We performed genotyping of mRNA sequences according to the Broad Institute’s Best Practices Workflow for RNAseq short variant discovery. Detailed descriptions of the genotyping and variant filtration workflow are provided in Additional file 3: Text S3. To characterize population genetic structure in both devils and DFTD tumors that may be associated with among-population variation in tumor gene expression, we performed separate discriminant analyses of principal components (DAPC), implemented in the R package *adegenet*, using the respective SNP datasets. We defined groups according to sample locality to characterize the extent of genetic differentiation among them; however, we also used the *find.clusters* function to infer genetic clusters purely from SNP variation, without a priori designation.

To identify specific SNPs and indels potentially associated with differentially expressed genes, we used SnpEff [47] to annotate all genotyped tumor variants according to their positions with respect to known genes (using the Ensembl v 7.0.86 annotation for *S. harrisii*) and quantifying each variant’s predicted impact on gene function. For example, a variant leading to loss or gain of a stop codon would have a predicted high impact, a nonsynonymous variant would be predicted to have a moderate impact, and a synonymous variant would be predicted to have a low impact [47]. We evaluated these annotations against our lists of differentially expressed genes to identify variants potentially contributing to or associated with up- or downregulation of genes. We tested all variants predicted to affect any gene that was differentially expressed between any pair of localities for their effects on expression of their respective genes (measured as counts per million and normalized as above) using a Kruskal-Wallis test. *P*-values were adjusted for multiple comparisons using the Benjamini-Hochberg approach, with an FDR threshold of 0.05.

## Supplementary Information

**Additional file 1 Table S1.** Sample metadata.

**Additional file 2 Table S2.** Differential gene expression contrasts performed for each analysis set.

**Additional file 3 Text S3.** Genotyping and variant filtration workflow for RNAseq data.

**Additional file 4 Fig. S4.** MDS plots showing expression variation with respect to sequencing batch for each analysis. Left-hand plots show batch effects prior to correction, while right-hand plots show batch-corrected expression variation.

**Additional file 5 Fig. S5.** Heat map indicating relative expression of 14,807 genes among 19 DFTD tumor samples and 39 normal lip tissues from Tasmanian devils (19 DFTD-infected, 20 putatively uninfected). Samples are arranged as columns, with genes arranged as rows. Differential expression analyses additionally accounted for sex, locality, batch, and tissue type – indicated for each sample as colored bars above each column. Relative gene expression is shown as a gradation from red (overexpressed) to blue (underexpressed). Dendrograms indicate clustering of samples (top) and genes (right) by similarity of expression.

**Additional file 6 Fig. S6.** Genes differentially expressed in DFTD tumors relative to normal lip tissues are enriched for various biological functions (GO-terms). Enriched biological functions are shown for genes up- and downregulated in DFTD, including functions that were enriched for both. Log2(fold enrichment) > 0 indicates overrepresented functions, while < 0 indicates underrepresented functions.

**Additional file 7 Fig. S7.** Venn diagram showing differentially expressed genes shared between each between-locality contrast among DFTD tumors.

**Additional file 8 S8 File.** .xlsx file containing tables of gene lists for all differential expression contrasts.

**Additional file 9 S9 File.** .xlsx file containing tables of significantly over- and underrepresented biological processes for up- and downregulated genes for each differential expression contrast.

**Additional file 10 S10 File.** .xlsx file containing tables of gene set enrichment results, including annotated gene set clusters.

**Additional file 11 S11 File.** .xlsx file containing tables of gene lists for all differential expression contrasts using batch 3 samples only.

**Additional file 12 S12 Table.** Table of results for GATK DepthOfCoverage analysis of sequencing coverage across different genomic regions: exons, 3’UTR, 5’UTR, < 5 kb downstream, < 5 kb upstream, introns, and intergenic regions. Total length of each region is provided, along with mean coverage across each region, as well as the percentage of each region covered at several coverage thresholds: 1x, 3x, 5x, and 10x.

**Additional file 13 S13 Table.** List of gene symbols mentioned in the manuscript text, with full gene names and short descriptions of each summarized from GeneCards.

## Data Availability

All sequence data are available at the NCBI Sequence Read Archive under BioProject PRJNA693818 (https://www.ncbi.nlm.nih.gov/bioproject/?term=PRJNA693818). All other sample data are provided in the Supplementary Materials.
