# Supplementary material for: Spatial variation in gene expression of Tasmanian devil facial tumors despite minimal host transcriptomic response to infection
Source: BMC Genomics. 2021 Sep 27;22:698. doi: 10.1186/s12864-021-07994-4 (PMC8477496; doi:10.1186/s12864-021-07994-4)
Supplement: Supplementary file 1 — Additional file 1 Table S1. Sample metadata. [file 12864_2021_7994_MOESM1_ESM.pdf]

S1 Table. Sample metadata.

Microchip	Tissue	Infected	Sex	Locality	Collection date	Batch	Tumor volume (mm^3)
982000402222562	DFTD	NA	F	BR	2016-12-01	2	3519
943094320451207	DFTD	NA	M	BR	2016-11-25	2	2420
982009106489786	DFTD	NA	F	TKN	2016-05-01	2	12582
982000363006955	DFTD	NA	F	TKN	2016-05-01	2	14422
982000167807486	DFTD	NA	F	TKN	2016-05-01	2	43798
943094320388439	DFTD	NA	M	BR	2016-12-02	2	83685
982000402218583	DFTD	NA	M	BR	2018-08-15	3	31638
943094320495423	DFTD	NA	F	BR	2018-05-03	3	5947
991001001626434	DFTD	NA	F	BR	2018-11-20	3	1066
982000356572975	DFTD	NA	M	TKN	2018-11-05	3	1099
982000405797085	DFTD	NA	M	TKN	2018-05-08	3	2343
982000405794197	DFTD	NA	M	TKN	2018-08-04	3	23385
982000405797607	DFTD	NA	F	TKN	2018-05-07	3	1848
982000405827172	DFTD	NA	M	WPP	2018-05-26	3	209021
982000365601031	DFTD	NA	M	WPP	2018-11-15	3	26678
982000405916991	DFTD	NA	M	WPP	2018-11-12	3	13935
982000365112018	DFTD	NA	F	WPP	2018-11-16	3	30504
982000365112172	DFTD	NA	F	WPP	2018-08-01	3	6035
982000405915086	DFTD	NA	F	WPP	2018-11-14	3	23674
943094320388439	lip	infected	M	BR	2016-12-02	2	NA
943094320451207	lip	infected	M	BR	2016-11-25	2	NA
982000167807486	lip	infected	F	TKN	2016-05-01	2	NA
982009106489786	lip	infected	F	TKN	2016-05-01	2	NA
982000363006955	lip	infected	F	TKN	2016-05-01	2	NA
982000402222562	lip	infected	F	BR	2016-12-01	2	NA
982000402218583	lip	infected	M	BR	2018-08-15	3	NA
943094320495423	lip	infected	F	BR	2018-05-03	3	NA
991001001626434	lip	infected	F	BR	2018-11-20	3	NA
982000405797085	lip	infected	M	TKN	2018-05-08	3	NA
982000356572975	lip	infected	M	TKN	2018-11-05	3	NA
982000405794197	lip	infected	M	TKN	2018-08-04	3	NA
982000405797607	lip	infected	F	TKN	2018-05-07	3	NA
982000405827172	lip	infected	M	WPP	2018-05-26	3	NA
982000405916991	lip	infected	M	WPP	2018-11-12	3	NA
982000365601031	lip	infected	M	WPP	2018-11-15	3	NA
982000365112172	lip	infected	F	WPP	2018-08-01	3	NA
982000365112018	lip	infected	F	WPP	2018-11-16	3	NA
982000405915086	lip	infected	F	WPP	2018-11-14	3	NA
982000365112065	lip	uninfected	F	BR	2016-06-29	1	NA

943094320451428	lip	uninfected	M	BR	2016-11-29	1	NA
982000356570815	lip	uninfected	M	TKN	2016-05-01	1	NA
982000365120402	lip	uninfected	M	BR	2016-12-01	1	NA
982000405915446	lip	uninfected	M	BR	2018-05-12	3	NA
982000402221837	lip	uninfected	F	BR	2018-05-08	3	NA
982000365594510	lip	uninfected	F	BR	2018-05-09	3	NA
982000405794482	lip	uninfected	M	TKN	2018-05-12	3	NA
982000405794197	lip	uninfected	M	TKN	2018-05-12	3	NA
982000410418505	lip	uninfected	M	TKN	2018-08-08	3	NA
900164001721621	lip	uninfected	M	TKN	2018-11-05	3	NA
982000356429856	lip	uninfected	F	TKN	2018-05-05	3	NA
982000405852285	lip	uninfected	F	TKN	2018-05-01	3	NA
982000410416717	lip	uninfected	F	TKN	2018-08-06	3	NA
982000405794412	lip	uninfected	M	WPP	2018-11-18	3	NA
982000365595034	lip	uninfected	M	WPP	2018-08-01	3	NA
982000365118323	lip	uninfected	M	WPP	2018-11-18	3	NA
982000365111978	lip	uninfected	F	WPP	2018-05-23	3	NA
982000405794419	lip	uninfected	F	WPP	2018-05-01	3	NA
900164001721631	lip	uninfected	F	WPP	2018-11-12	3	NA
