# Supplementary material for: Spatial variation in gene expression of Tasmanian devil facial tumors despite minimal host transcriptomic response to infection
Source: BMC Genomics. 2021 Sep 27;22:698. doi: 10.1186/s12864-021-07994-4 (PMC8477496; doi:10.1186/s12864-021-07994-4)
Supplement: Supplementary file 2 — Additional file 2 Table S2. Differential gene expression contrasts performed for each analysis set. [file 12864_2021_7994_MOESM2_ESM.pdf]

S2 Table. Differential expression contrasts. Differential gene expression contrasts performed for each analysis set.

Analysis set	Contrast
Tissue comparison	
	all.DFTD - all.lip
	BR.DFTD - BR.lip
	TKN.DFTD - TKN.lip
	WPP.DFTD - WPP.lip
Lip-only	
	all.infected - all.uninfected
	female.infected - female.uninfected
	male.infected - male.uninfected
	all.male - all.female
	infected.male - infected.female
	uninfected.male - uninfected.female
	all.WPP - all.TKN
	all.TKN - all.BR
	all.BR - all.WPP
	(male.infected - male.uninfected) - (female.infected - female.uninfected)
DFTD-only	
	all.male - all.female
	BR.male - BR.female
	TKN.male - TKN.female
	WPP.male - WPP.female
	all.WPP - all.TKN
	all.TKN - all.BR
	all.BR - all.WPP
