# Supplementary material for: Spatial variation in gene expression of Tasmanian devil facial tumors despite minimal host transcriptomic response to infection
Source: BMC Genomics. 2021 Sep 27;22:698. doi: 10.1186/s12864-021-07994-4 (PMC8477496; doi:10.1186/s12864-021-07994-4)
Supplement: Supplementary file 3 — Additional file 3 Text S3. Genotyping and variant filtration workflow for RNAseq data. [file 12864_2021_7994_MOESM3_ESM.pdf]

S3 Text. Genotyping and variant filtration workflow for RNAseq data.

First, we aligned raw sequence reads to the devil reference genome using STAR (Dobin et al., 2013) with the multi-sample 2-pass mapping approach to optimize sequence alignment around novel splice junctions. The remaining steps were performed separately for lip and tumor samples using functions from the Broad Institute's software packages Genome Analysis Toolkit (GATK) v 4.1.4 and Picard Tools v 4.1.4. We used the *MarkDuplicates* function to identify duplicate reads for removal, followed by *SplitNCigarReads* to split reads that span introns. For each sample, we performed base quality score recalibration (BQSR) using *BaseRecalibator* and *ApplyBQSR*, which correct patterns of systematic error in base quality scores by analyzing mismatches between sequence reads and the reference and recalibrating base quality scores according to various covariates. This approach is designed for model organisms and thus relies on a database of known polymorphic sites to be masked out, with mismatches at non-masked sites regarded as errors and indicative of poor base quality. In lieu of a sufficiently comprehensive database, we performed a bootstrapping procedure to generate this database from our own sequences. Specifically, we first ran the entire genotyping workflow (including further steps below) without BQSR and used the resulting SNPs as a database of known sites for BQSR in a subsequent run. This was repeated several times, using *AnalyzeCovariates* to evaluate successive BQSR runs, until convergence. Following BQSR, we used *HaplotypeCaller* to call variants and produce an intermediate GVCF file per sample, followed by joint genotyping using *GenotypeGVCFs* to produce a joint VCF file. We used *SelectVariants* to separate SNPs and indels and *VariantFiltration* to apply a hard filter to each. For hard filtering, we used a cluster size of 3 SNPs across a 35 bp window, removing SNPs with a quality by depth (QD) < 5.0, Fisher strand bias (FS) > 60.0, strand odds ratio (SOR) > 3.0, root mean square of the mapping quality (MQ) < 40.0, mapping quality rank sum test (MQRankSum) < -12.5, or a read position rank sum test

(ReadPosRankSum) < -5.0 or > 5.0, and removing indels with QD < 2.0, FS > 200.0, SOR > 10.0, and -5.0 > ReadPosRankSum > 5.0.

Following hard filtering, we used the isec function in bcftools to exclude known devil germline variants from the DFTD tumor dataset. Our set of known germline variants included those genotyped in the present study, as well as those genotyped from 10 high coverage whole genome libraries described in Margres et al. (2018). We used Vcftools to apply additional quality filters to each dataset separately. Both lip and tumor variants were filtered to a minimum allele depth of 3, minimum quality score of 20, and a minimum minor allele count of 3. Variants were additionally filtered to include only those genotyped in > 70% of samples for lips and > 50% of samples for tumors.

References

Dobin, A., Davis, C. A., Schlesinger, F., Drenkow, J., Zaleski, C., Jha, S., … Gingeras, T. R. (2013). STAR: ultrafast universal RNA-seq aligner. *Bioinformatics*, *29*(1), 15–21. doi: 10.1093/bioinformatics/bts635

Margres, M. J., Ruiz-Aravena, M., Hamede, R., Jones, M. E., Lawrance, M. F., Hendricks, S. A., … Storfer, A. (2018). The Genomic Basis of Tumor Regression in Tasmanian Devils (Sarcophilus harrisii). *Genome Biology and Evolution*, *10*(11), 3012–3025. doi: 10.1093/gbe/evy229
