# Supplementary material for: Spatial variation in gene expression of Tasmanian devil facial tumors despite minimal host transcriptomic response to infection
Source: BMC Genomics. 2021 Sep 27;22:698. doi: 10.1186/s12864-021-07994-4 (PMC8477496; doi:10.1186/s12864-021-07994-4)
Supplement: Supplementary file 4 — Additional file 4 Fig. S4. MDS plots showing expression variation with respect to sequencing batch for each analysis. Left-hand plots show batch effects prior to correction, while right-hand plots show batch-corrected expression variation. [file 12864_2021_7994_MOESM4_ESM.pdf]

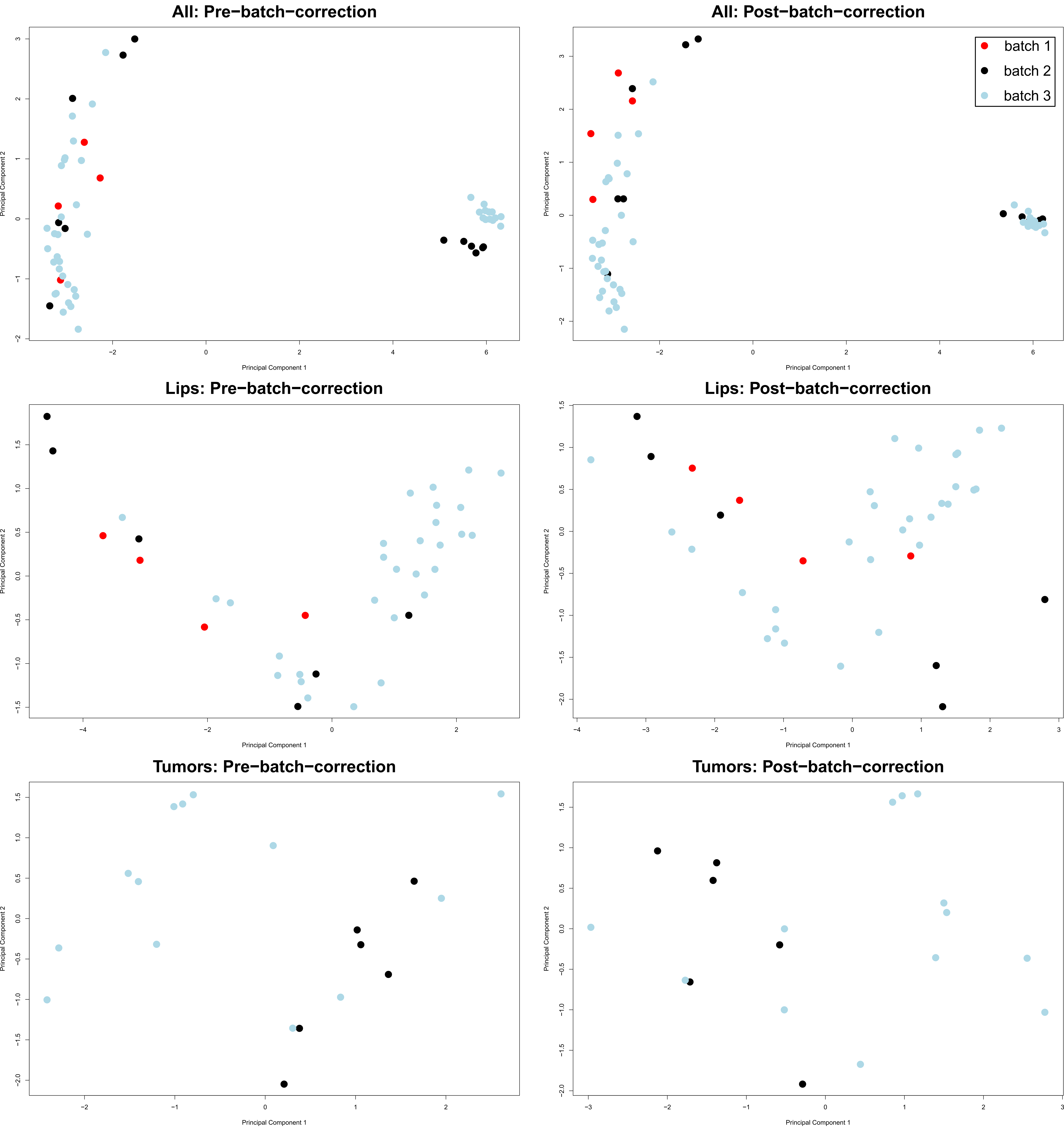
All: Pre−batch−correction
All: Post−batch−correction
batch 1
batch 2
batch 3
Lips: Pre−batch−correction
Lips: Post−batch−correction
Tumors: Pre−batch−correction
Tumors: Post−batch−correction
Principal Component 1
Principal Component 2
