# Supplementary figures and images for: Spatial variation in gene expression of Tasmanian devil facial tumors despite minimal host transcriptomic response to infection
Source: BMC Genomics. 2021 Sep 27;22:698. doi: 10.1186/s12864-021-07994-4 (PMC8477496; doi:10.1186/s12864-021-07994-4)

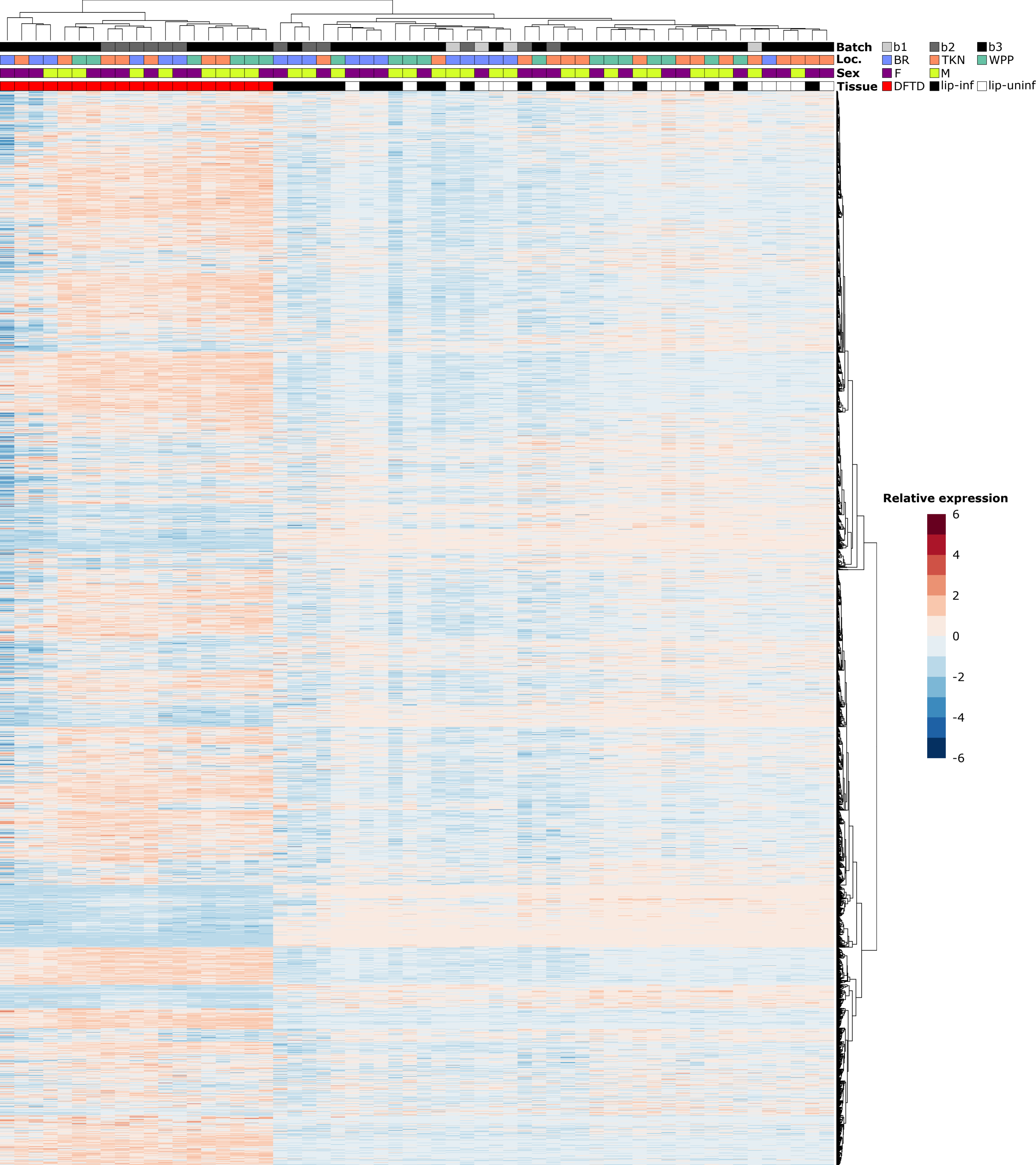

Supplement: Supplementary file 5 — Additional file 5 Fig. S5. Heat map indicating relative expression of 14,807 genes among 19 DFTD tumor samples and 39 normal lip tissues from Tasmanian devils (19 DFTD-infected, 20 putatively uninfected). Samples are arranged as columns, with genes arranged as rows. Differential expression analyses additionally accounted for sex, locality, batch, and tissue type – indicated for each sample as colored bars above each column. Relative gene expression is shown as a gradation from red (overexpressed) to blue (underexpressed). Dendrograms indicate clustering of samples (top) and genes (right) by similarity of expression. [file 12864_2021_7994_MOESM5_ESM.pdf]

GO-term

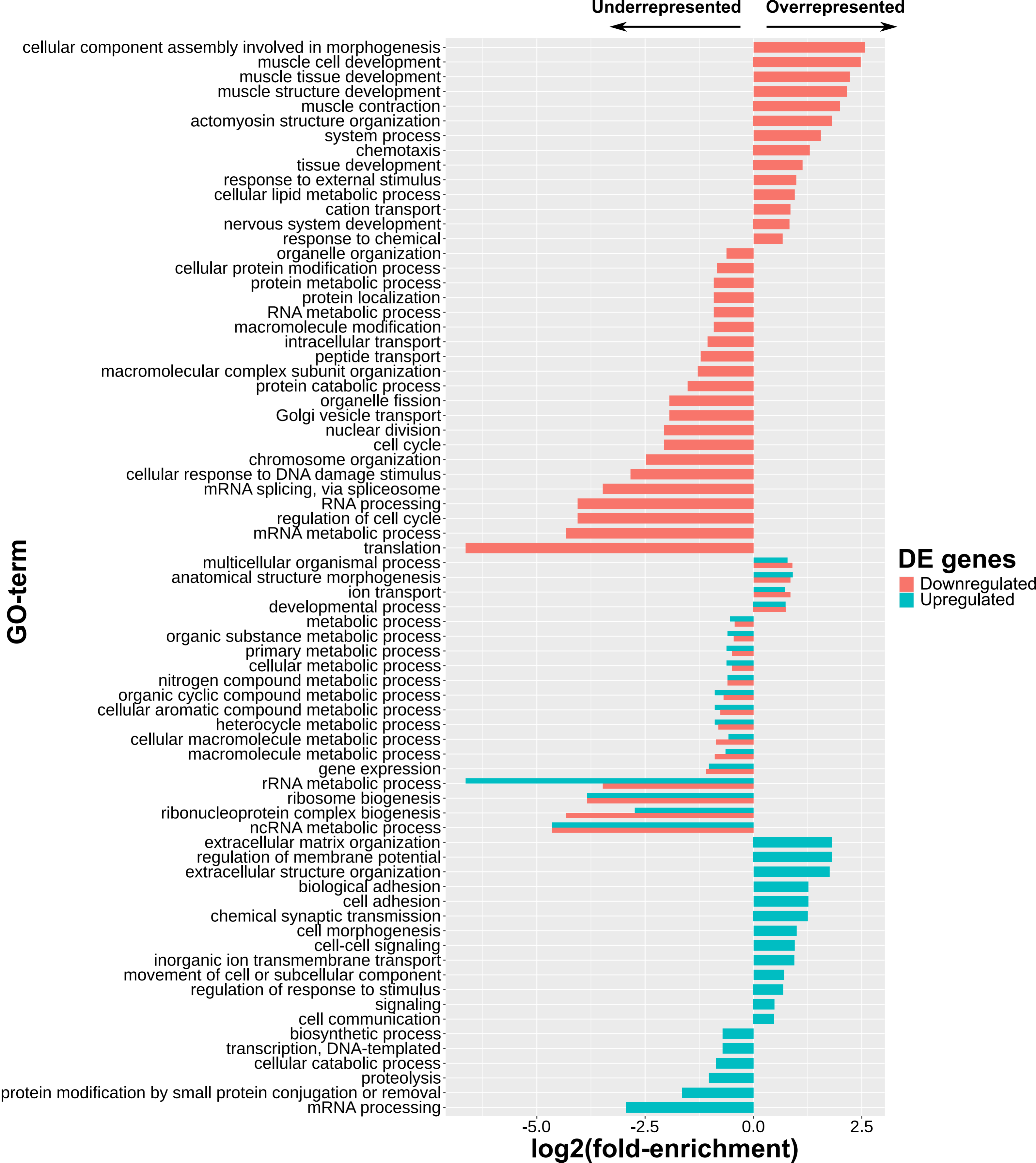

Supplement: Supplementary file 6 — Additional file 6 Fig. S6. Genes differentially expressed in DFTD tumors relative to normal lip tissues are enriched for various biological functions (GO-terms). Enriched biological functions are shown for genes up- and downregulated in DFTD, including functions that were enriched for both. Log2(fold enrichment) > 0 indicates overrepresented functions, while < 0 indicates underrepresented functions. [file 12864_2021_7994_MOESM6_ESM.pdf]

**BR vs. WPP**

**TKN vs. BR**

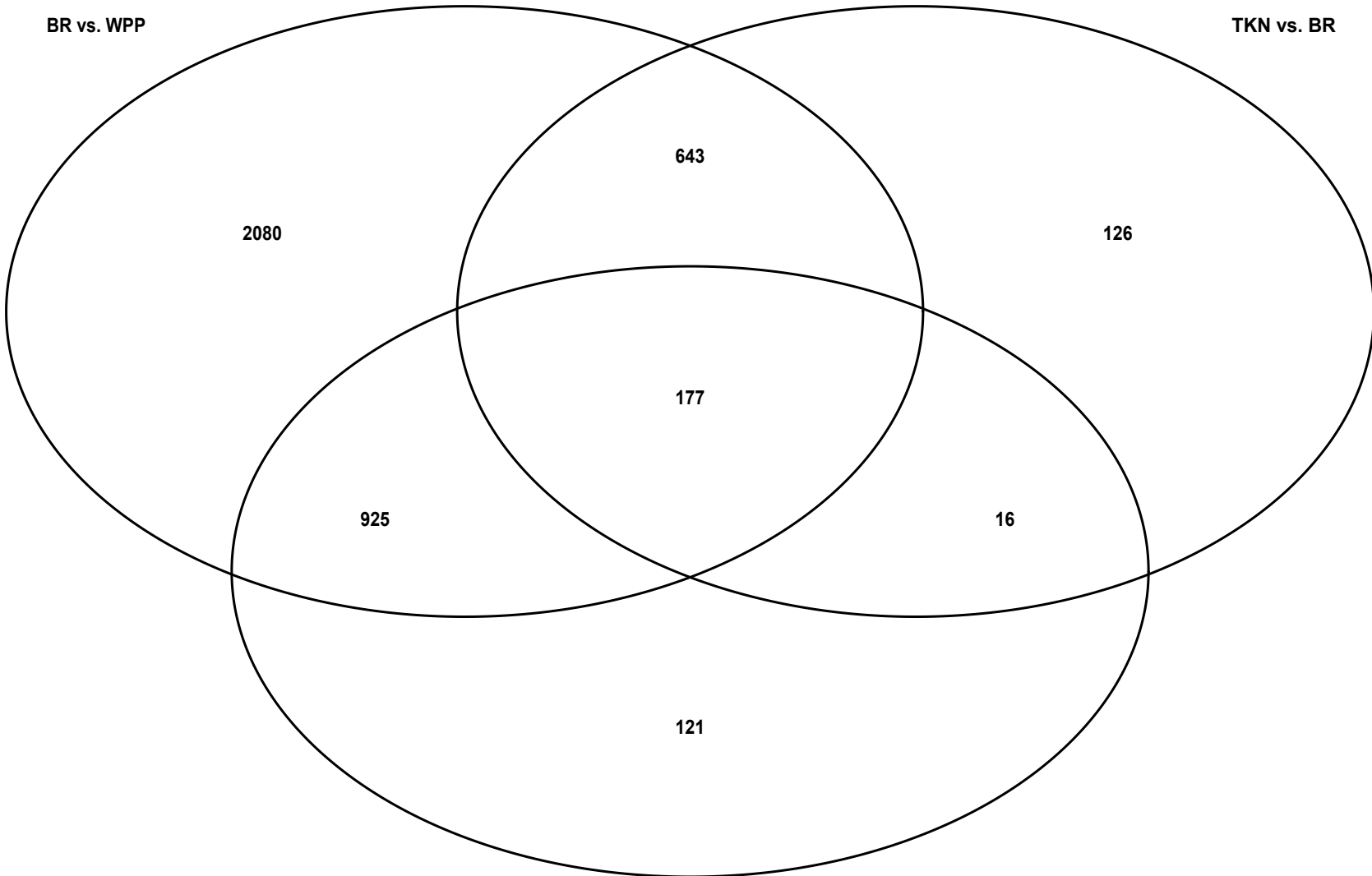

**WPP vs. TKN**

Supplement: Supplementary file 7 — Additional file 7 Fig. S7. Venn diagram showing differentially expressed genes shared between each between-locality contrast among DFTD tumors. [file 12864_2021_7994_MOESM7_ESM.pdf]
