# Supplementary material for: Spatial variation in gene expression of Tasmanian devil facial tumors despite minimal host transcriptomic response to infection
Source: BMC Genomics. 2021 Sep 27;22:698. doi: 10.1186/s12864-021-07994-4 (PMC8477496; doi:10.1186/s12864-021-07994-4)
Supplement: Supplementary file 12 — Additional file 12 S12 Table. Table of results for GATK DepthOfCoverage analysis of sequencing coverage across different genomic regions: exons, 3’UTR, 5’UTR, < 5 kb downstream, < 5 kb upstream, introns, and intergenic regions. Total length of each region is provided, along with mean coverage across each region, as well as the percentage of each region covered at several coverage thresholds: 1x, 3x, 5x, and 10x. [file 12864_2021_7994_MOESM12_ESM.pdf]

S12 Table. Sequencing coverage per genomic region

LIP	Total length (bp)	Mean coverage*	% bases > 1x*	% bases > 3x*	% bases > 5x*	% bases > 10x*
**3UTR**	8,152,227	25.24 ± 8.82	52.20 ± 2.35	39.44 ± 2.34	33.47 ± 2.31	25.56 ± 2.42
**5UTR**	2,327,236	9.67 ± 2.99	34.37 ± 1.51	23.60 ± 1.83	18.87 ± 2.02	13.04 ± 2.18
**Coding exons**	29,265,941	46.26 ± 13.34	65.21 ± 2.17	55.11 ± 2.71	49.00 ± 3.24	39.18 ± 4.22
**Downstream**	96,653,345	8.44 ± 2.62	24.58 ± 1.67	16.13 ± 1.05	12.91 ± 0.95	9.19 ± 0.98
**Intergenic**	2,648,837,973	0.64 ± 0.16	2.68 ± 0.49	1.22 ± 0.14	0.89 ± 0.08	0.61 ± 0.08
**Introns**	666,579,587	0.26 ± 0.04	4.70 ± 1.76	1.33 ± 0.50	0.69 ± 0.24	0.31 ± 0.06
**Upstream**	100,392,563	2.47 ± 0.65	8.52 ± 1.37	3.83 ± 0.40	2.68 ± 0.23	1.73 ± 0.19
**TUMOR**	**Total length (bp)**	**Mean coverage***	**% bases > 1x***	**% bases > 3x***	**% bases > 5x***	**% bases > 10x***
**3UTR**	8,152,227	23.97 ± 9.20	54.46 ± 3.60	41.78 ± 3.95	35.86 ± 4.18	27.84 ± 4.45
**5UTR**	2,327,236	10.87 ± 4.90	37.71 ± 5.44	26.11 ± 5.50	21.07 ± 5.34	14.76 ± 4.63
**Coding exons**	29,265,941	40.58 ± 17.35	66.30 ± 5.60	56.61 ± 8.07	50.80 ± 9.33	41.27 ± 10.40
**Downstream**	96,653,345	8.44 ± 3.47	28.11 ± 3.45	18.62 ± 2.91	14.96 ± 2.68	10.73 ± 2.40
**Intergenic**	2,648,837,973	0.66 ± 0.23	4.32 ± 1.41	1.60 ± 0.41	1.10 ± 0.26	0.72 ± 0.17
**Introns**	666,579,587	0.36 ± 0.10	8.09 ± 3.78	2.01 ± 0.93	0.96 ± 0.38	0.40 ± 0.14
**Upstream**	100,392,563	2.27 ± 0.79	11.47 ± 2.98	4.86 ± 1.29	3.23 ± 0.83	1.98 ± 0.52

* mean calculated from subset of 9 samples ± standard deviations
