# Supplementary material for: Spatial variation in gene expression of Tasmanian devil facial tumors despite minimal host transcriptomic response to infection
Source: BMC Genomics. 2021 Sep 27;22:698. doi: 10.1186/s12864-021-07994-4 (PMC8477496; doi:10.1186/s12864-021-07994-4)
Supplement: Supplementary file 13 — Additional file 13 S13 Table. List of gene symbols mentioned in the manuscript text, with full gene names and short descriptions of each summarized from GeneCards. [file 12864_2021_7994_MOESM13_ESM.pdf]

S13 Table. Gene descriptions

Gene symbol	Full name	Description*
FRMD7	FERM Domain Containing 7	plays role in neurite development, eye movement, and gaze stability
HMGB3	High Mobility Group Box 3	DNA binding, maintenance of stem cell populations, potential role in innate immune response
MECP2	Methyl-CpG Binding Protein 2	chromatin binding, DNA methylation, transcription regulation
SYNE2	Spectrin Repeat Containing Nuclear Envelope Protein 2	actin-binding nuclear outer membrane protein
CLUH	Clustered Mitochondria Homolog	mRNA-binding, translation regulation, mitochondrial biogenesis
PARS2	Prolyl-TRNA Synthetase 2, Mitochondrial	protein biosynthesis
VPS13A	Vacuolar Protein Sorting 13 Homolog A	protein binding
KIAA1586	E3 SUMO-Protein Ligase KIAA1586	nucleic acid binding
TASOR2	Transcription Activation Suppressor Family Member 2	protein binding, associated with anal squamous cell carcinoma and anus cancer
SLC12A5	Solute Carrier Family 12 Member 5	ion transport, protein kinase binding, involved in neuron function
TRRAP	Transformation/Transcription Domain Associated Protein	transcription and DNA repair
DIP2A	Disco Interacting Protein 2 Homolog A	axon patterning in central nervous system
BRPF1	Bromodomain And PHD Finger Containing 1	transcriptional regulation, including of TP53 activity
PCNX3	Pecanex 3	transmembrane protein
MRPL53	Mitochondrial Ribosomal Protein L53	mitochondrial protein synthesis
TP53	Tumor Protein P53	tumor suppressor involved in cell cycle regulation
ERBB2	Erb-B2 Receptor Tyrosine Kinase 2	epidermal growth factor signaling; associated with various cancers
ERBB3	Erb-B2 Receptor Tyrosine Kinase 3	epidermal growth factor signaling; associated with various cancers
STAT3	Signal Transducer And Activator Of Transcription 3	mediates cellular responses to growth factors, involved in cell growth and apoptosis
MMP2	Matrix Metallopeptidase 2	zinc-dependent enzyme, degrades extracellular matrix proteins
HDAC5	Histone Deacetylase 5	transcription regulation, cell cycle progression, and development
PTGIS	Prostaglandin I2 Synthase	enzyme involved in lipid metabolism and biosynthesis
TRIM28	Tripartite Motif Containing 28	nucleic acid binding, transcription regulation
NRG1	Neuregulin 1	cell-cell signalling, organ development

* Gene descriptions summarized from GeneCards (https://www.genecards.org)
